# Automated Diagnosis of Hypertensive Retinopathy Using Res‐UNet and Graph Convolutional Networks

**DOI:** 10.1049/htl2.70054

**Published:** 2026-02-09

**Authors:** Esra'a Mahmoud Jamil AL Sariera

**Affiliations:** ^1^ Department of Computer Science Faculty of Information Technology Al‐Ahliyya Amman University Amman Jordan

**Keywords:** blood vessels, eye, feature extraction, health care, image classification, image processing, image segmentation, medical image processing

## Abstract

Hypertensive retinopathy (HR), a progressive retinal condition, is associated with both hypertension and diabetes mellitus. The development of HR is closely correlated with the severity and duration of hypertension. The results of the HR indicate that pathological eye issues include cotton‐like spots, macular oedema, constrained arterioles and retinal haemorrhage. An ophthalmologist would still often undertake a manual physical examination using an ophthalmoscope to detect HR in a patient's body. It is time‐consuming for a physician to identify HR in a patient based only on retina fundus imaging when done manually. An automated approach for identifying the retinal fundus image is required to solve this issue. One crucial component in the diagnosis of many eye diseases is the condition of the blood vessels in the retina. Researchers have found great interest in the blood vessel segmentation of fundus images for this reason. The knowledge of blood vessel changes associated with various disorders, such as cardiovascular diseases and retinopathy, depends on the accurate segmentation of arteries and veins (A/V) from fundus images. The arteriovenous ratio displays the proportion of vein to artery diameters. The precision with which vessels are divided into veins and arteries determines the significance of this measure. To increase the accuracy of classifying retinal blood vessels and HR phases, a novel technique combining deep residual UNET (Res‐UNet) and a graph convolutional network is suggested in this research. Pre‐processing (green channel, contrast‐restricted adaptive histogram equalisation) was done before identification. Graphs are used to depict the features of the vessel that are extracted from the spatial domain. The DRIVE‐AV image dataset is used to execute the proposed approach and the results show that the system achieves a blood vessel segmentation accuracy of 96.45% and an A/V classification of 96.7%.

## Graphical abstract

The proposed framework integrates retinal vessel segmentation and artery/vein classification using deep residual UNET and GCN models for hypertensive retinopathy diagnosis. By computing the arteriovenous ratio, the method accurately detects HR stages from retinal fundus images. The system achieves reliable performance using the DRIVE dataset.

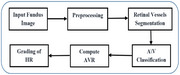

## 1 Introduction

**FIGURE 1:**
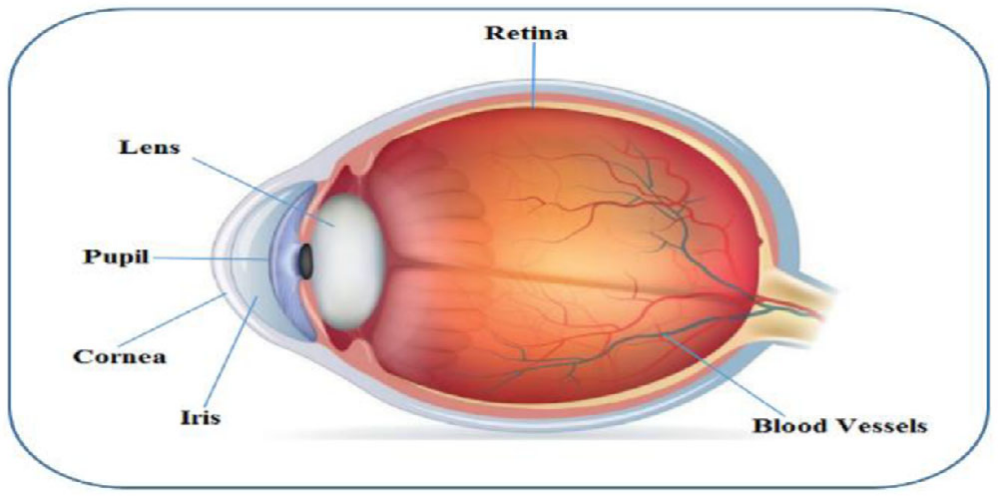
Anatomy of the human eye [2].

The significance of the eyes in the human body is attributed to their crucial function of providing us with the ability to perceive the visual stimuli necessary for observing and comprehending the surrounding environment. The back part of the eye is where the retina, a thin layer of membranous tissue, is situated and it is responsible for ensuring that our daily activities are carried out with sharp and clear vision. However, several retinal pathologies, including microaneurysms, diabetic retinopathy and HR, impair the retina as we age. The field of medical science has been working on early disease detection through retinal imaging. Three layers make up the human eye. The sclera, or outer layer, is what shields the eyeball. The choroid, which contains blood vessels and supplies nourishment to the whole eyeball, is located underneath the sclera. The retina receives the vision after it has passed through the pupil and the biconvex lens. On the fovea, this vision forms an inverse, scaled image [1]. The macular area, which surrounds the rod and cone cells in the fovea, has a diameter of around 5500 microns. The anatomy of the human eye is depicted in Figure 1.

**FIGURE 2:**
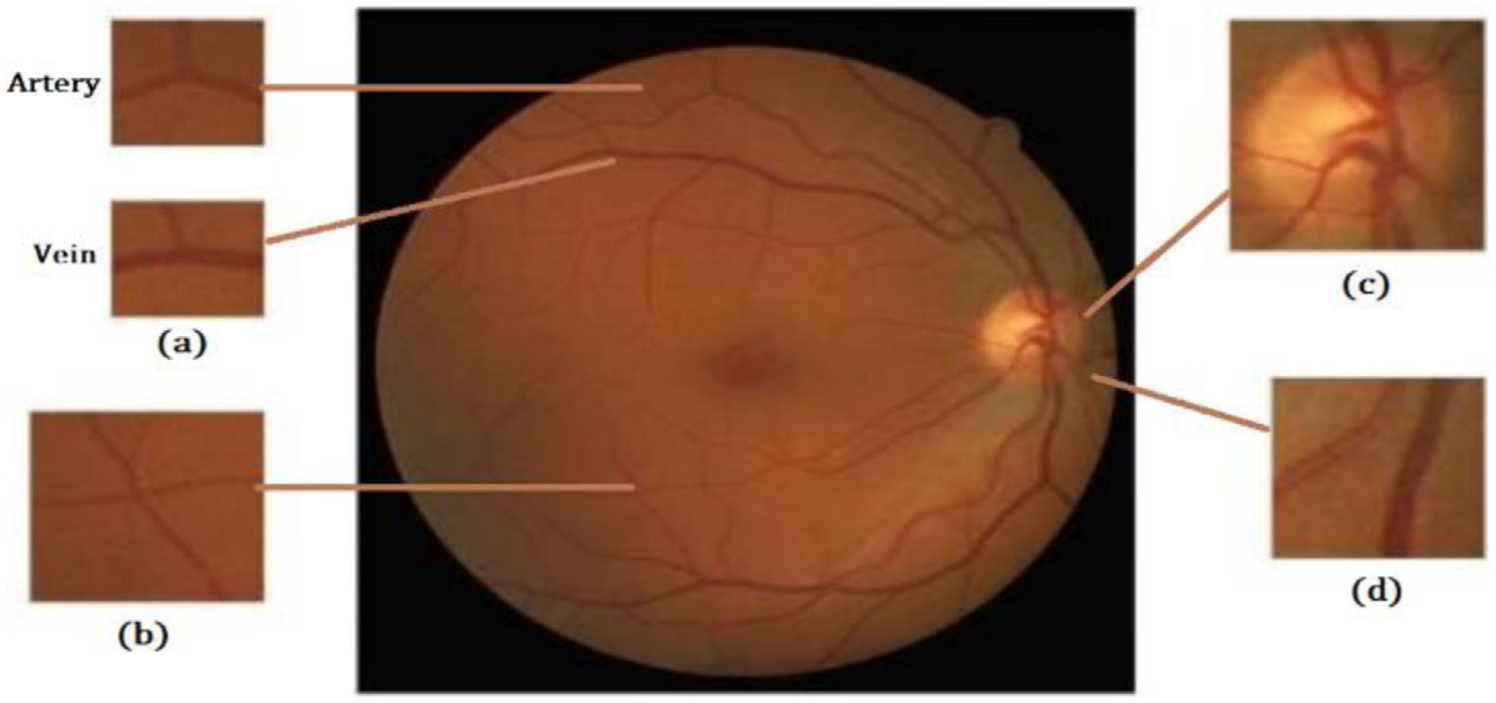
Vessels located across the retina: (a) main veins are darker than arteries;(b) thin veins and arteries share features. (c) The optical disk's interconnected vessels. (d) The central reflex is more apparent in veins than in arteries.

The retinal blood vessels in the eyes are made up of arteries and veins (A/V) that have a structure resembling a tree, roots and branches. The widths of the vascular can vary in different locations because of factors such as the central light reflex, background noise and false pixels due to the optic disc shadow. The arteries carry oxygenated blood, which is brighter, while the veins carry deoxygenated blood and appear darker. When a person has hypertension, their blood vessels can change, causing the arteries to thicken due to the elevated blood pressure. This can lead to a stroke, but the changes can also be seen regarding the structure or shape of the blood vessels within the retina. Additionally, hypertensive patients display an anomalous relationship between the average diameters of veins and arteries, resulting in an atypical ratio between them [3]. Figure 2 shows blood vessels in different parts of the retina.

Approximately 9.4 million people globally suffer from hypertension each year [4]. By 2025, 1.6 billion people worldwide are predicted to have hypertension [5]. Hypertension causes damage to blood vessels and the retina. Hypertension can take many different forms and affect every part of the body. This occurs as a result of blood pressure that is higher than usual and may harm the retina. Hypertensive retinopathy (HR) is the term for retinal impairment brought on by hypertension [6]. HR can cause long‐term damage to the retina because high blood pressure has accumulated in the eye for several years, even in patients who control their blood pressure with drugs [7]. Existing research showed that 25.8% of total victims are people aged over 18 and that men have a higher possibility of having hypertension in productive years. However, this does not apply to people in their 50s or above, since in this age range of age, women tend to have a higher rate of possibility of experiencing this disease. Early identification of HR is very important to protect the retina from these diseases. Many hypertensive patients have been diagnosed with these diseases caused by HR. The majority of people lose their vision when HR symptoms develop [8]. Several studies have revealed that using a fundus digital camera, retinal microvascular variations may be observed [9]. This form of imaging is known for its user‐friendly nature and its capability to display the majority of anatomical structures related to lesions. Ophthalmologists use a four‐level categorisation system to classify hypertensive retinopathy. The first level is identified by a slight narrowing of the retinal arteriolar patterns, while the second level is characterised by a more significant reduction in the retinal artery, known as arteriovenous nipping, along with symptoms from the first level. The third level is considered an advanced stage and features the presence of hard exudates, cotton wool spots, microaneurysms and retinal haemorrhages on the fundus of the retina, as well as signs from the second level. The fourth and most severe level of hypertensive retinopathy is identified by the swelling and blurring of the optic disc, which is an indication of papilledema. Individuals with this level of hypertensive retinopathy have a much higher risk of stroke and cardiovascular disease, in addition to displaying symptoms from the third level. The classification of A/V is of most importance in the precise diagnosis of cardiovascular ailments and the evaluation of the development of HR. Accurate classification of these medical data can assist healthcare professionals in identifying potential health concerns and providing appropriate treatments. Several methods have been suggested for the classification of A/V, including traditional blood vessel classification methods and modern CNN models. However, topological models that follow conventional techniques face limitations in their ability to make efficient use of deep hierarchical features extracted by CNN, whereas CNN‐based methods in the present times encounter difficulties in integrating vascular topology information. Therefore, in this study, we introduce a new and innovative approach that combines the Res‐UNet and GCN to enhance the precision of categorising vascular A/V. So first, we convert the vessel features extracted from the Res‐UNet into a GCN. Finally, the images are classified as either normal or abnormal.

The remainder of this paper is organised as follows. Section 2 reviews the most relevant literature on retinal vessel segmentation, artery/vein classification and hypertensive retinopathy analysis. Section 3 presents the proposed methodology in detail, including image preprocessing, the Res‐UNet segmentation framework, the graph‐based artery/vein classification module and the computation of AVR. Section 4 reports the experimental setup and evaluation results, followed by a comprehensive discussion and comparative analysis with existing methods. Section 5 summarises the main findings of the study and outlines potential directions for future research.

## 2 Related Work

### 2.1 Segmentation of Blood Vessels

Numerous techniques have been effectively used to diagnose medical images [10, 11] and to segment blood vessels in the retinal images and categorise them as either veins or arteries. In [12], the researchers came up with a two‐stage method that involves using a CNN in the initial phase to establish a connection between the image and the corresponding ground truth through random tree embedding. Subsequently, in the second stage, the CNN is fed with training patches to form a codebook, which is used to construct a generative nearest‐neighbour search space for the feature vector. A new technique, known as cross‐connected CNN (CcNet), was proposed to segment retinal vessels. The researchers trained the CcNet using solely the green channel of the fundus image and enhanced the network efficacy by including cross‐connections and multi‐scale feature merging [13]. Mishra et al. [14] introduced a novel framework called VTG‐Net, which stands for vascular architecture for enhancing the precision of categorising retinal blood vessels into arteries or veins classification. This framework incorporates vessel architecture information along with features extracted by CNN to improve classification accuracy. The method involves transforming vessel features extracted from the retinal image using a CNN by converting the retinal vasculature into a graph that retains its vascular architecture. The fully convolutional network was set up by Xu et al. [15] to accept multiple labels as output and real colour images as input and suggested the use of an enhanced FCN architecture to segment retinal arterioles and venules simultaneously. End‐to‐end multi‐label segmentation of a colour fundus image was made possible by this technique. A loss function that is particular to a given domain was created to enhance overall performance. To segment the whole retinal vessel, artery and vein at the same time, Ma et al. [16] present a multi‐task deep neural network with a spatial activation mechanism that does not need vascular segmentation beforehand. Furthermore, deep supervision is added to the network to help the lower‐level layers extract additional semantic data. [17] provide a unique technique that, compared to other methods, breaks down the joint work into three segmentation tasks that target arteries, veins and the entire vascular tree. This is made possible by the suggestion of a novel loss. This approach immediately delivers accurate segmentation masks of the various target vascular trees and enables straightforward handling of vessel crossings. More vessels can be detected and better segmentation of the various structures is possible with the suggested multi‐segmentation approach.

### 2.2 Classification of Artery/ Vein

Numerous techniques have been used to classify arteries/veins in retinal images. Welikala et al. [18] employed a six‐layer CNN to learn features from the vessels to classify AV crossings. Zhao et al. [19] developed a graph to classify A/V in retinal images by using image segmentation, skeletonisation and the recognition of relevant nodes. The researchers defined the pairwise clustering challenge from the topology estimation and artery/vein classification. Xu et al. [20] An enhanced supervised technique for classifying veins and arteries in retinal images is presented in this research. To reduce the variations in feature space, intra‐image regularisation and inter‐subject normalisation are used. First‐ and second‐order texture features, among other novel aspects, are used to capture the distinctive properties of veins and arteries. Zhao and associates [21] presented a novel approach for classifying veins and arteries based on vascular topological features. To precisely analyse the retinal vasculature, it blends DOS with graph‐theoretic techniques. The process of building the graph involves segmenting the image, skeletonising it and identifying the important nodes. The definition of the edge weight in the feature space of intensity, orientation, curvature, diameter and entropy is the inverse Euclidean distance between its two endpoints. Based on its intensity and shape, the rebuilt vascular network is divided into A/V [22]. In this study, a novel graph search metaheuristic technique for automated A/V separation from colour fundus images is provided. The technique makes use of global information to classify these vessel sub‐trees as A/V and local information to break up the intricate vascular tree into many sub‐trees. A graph representation of the vascular network, which depicts the topological and spatial interconnectedness of the vascular structures, is created using a binary vessel map. The vascular tree is divided into several subtrees, including A/V, according to the anatomical uniqueness at artery crossing and branching locations. Lastly, an A/V label is applied to the detected vessel sub‐trees using a set of manually created features that were learned using a random forest classifier. Kang et al. [23] presented AVNet, a new segmentation network that integrates the category‐attention weighted fusion module to greatly improve the pixel‐level A/V classification results. This successfully improves the model's classification performance. The impact of the graph model on the noisy vessel segmentation results is then confirmed by using a graph‐based vascular structure reconstruction approach to reduce the segment‐wise inconsistency [24]. In combination with a post‐processing technique, this research presented SeqNet for precise vascular segmentation and artery/vein classification in retinal images. Due to the uneven label distribution issue, SeqNet performs segmentation and classification in a sequential manner rather than concurrently, which might lead to a decline in segmentation performance. The classification results are subsequently corrected by the post‐processing method by propagating highly confident labels to the surrounding vessel segments. A multiscale guided attention network called MSGANet‐RAV is proposed by Chowdhury et al. [25] for pixel‐wise retinal artery‐vein classification. The suggested architecture combined a series of GF and context‐learnable SVA modules with multi‐scale feature learning.

Accurate differentiation between retinal A/V is fundamental for reliable A/V classification, AVR computation and hypertensive retinopathy assessment. Although both vessel types share structural similarities, they exhibit distinct anatomical, morphological and physiological characteristics that influence their appearance in fundus images and their behaviour under preprocessing and segmentation operations. To provide a clearer context and support the rationale for the proposed graph‐based A/V classification framework, Table 1 summarises the key distinguishing features between retinal A/V.

**TABLE 1:** Comparison between retinal arteries and veins.

Feature	Retinal arteries	Retinal veins
Colour/intensity	Brighter, lighter red due to higher oxygenation	Darker, deeper red or purplish tint
Vessel width	Narrower than veins	Wider lumen; generally thicker
Central reflex (light reflex)	Strong, distinct central light reflex	Weak or absent central reflex
Branching pattern	More acute branching angles; sharper bifurcation	Larger branching angles; smoother bifurcation
Wall structure/edge sharpness	Sharper boundaries, more well‐defined edges	Softer boundaries and slightly blurred edges
Tortuosity	Less tortuous	More torturous in many cases
Physiological role	Carry oxygenated blood from the optic disc to the retina	Return deoxygenated blood from the retina to the optic disc
Response to hypertension	Constriction → ↓ CRAE	Dilation → ↑ CRVE
Impact on AVR (artery‐to‐vein ratio)	Reduced artery width decreases AVR	Enlarged vein width decreases AVR
Appearance under CLAHE/preprocessing	Enhanced brightness and contour visibility	Appears darker even after enhancement
Crossing behaviour	At the A‐V crossing, the artery typically crosses over the vein	Veins usually appear underneath the artery
Sensitivity to pathologies	Shows early narrowing in hypertensive retinopathy	Shows dilation and engorgement in late HR stages
Common classification challenges	Very thin arteries may resemble capillaries	Wide veins in diseased images may resemble arteries
Topological behaviour in graph models	Higher centrality near arterial trees	More distributed, lower edge centrality
Segmentation behaviour	Often segmented as thinner structures	Segmented as thicker, elongated structures
Vessel width	Narrower than veins	Wider lumen; generally thicker
Central reflex (light reflex)	Strong, distinct central light reflex	Weak or absent central reflex

### 2.3 Classification of HR

Numerous techniques have been used to classify HR in retinal images. Arsalan et al. [26] created the Arsalan‐HR system, which was created to identify blood vessels in fundus images by employing a dual residual path technique. Utilising semantic segmentation, the researchers were able to differentiate between HR and non‐HR stages within a deep learning framework that required minimal parameters. In a recent publication [27], a Tang‐Semantic system was devised by researchers using semantic segmentation through a CNN architecture. This method aims to detect and locate lesions associated with diabetic retinopathy (DR); however, the AVR ratio was not taken into consideration in the author's analyses and they only identified DR‐related lesions. It has been observed that CNNs may misclassify certain cases that appear to be straightforward for graph‐based methods, possibly due to the limitations of their feature extractors in capturing vessel architecture accurately. Therefore, it is thought that integrating a CNN‐based technique with a deep graph‐based model capable of accurately representing vessel architecture could improve A/V categorisation accuracy [28]. This study develops a novel five‐grade hypertensive retinopathy (HYPER‐RETINO) framework for grading HR. Based on previously trained HR‐related lesions, the HYPER‐RETINO system is put into use. Several procedures are used in the development of this HYPER‐RETINO system, including preprocessing, semantic and instance‐based segmentation for the identification of HR‐related lesions and the use of a DenseNet architecture for HR stage classification. To identify five classes of HR, the HYPER‐RETINO algorithm identified local areas within input retinal fundus images [29]. To improve A/V classification and overcome the aforementioned restrictions, this research offers a unique multi‐task segmentation and classification network that makes use of the vessel features retrieved by a particular module. The proposed method combines three modules to improve the performance of A/V classification: multi‐structure A/V extraction classifies A/V by combining the original image with the vessel features produced by the multi‐scale vessel extraction (MVE) module; MVE separates vessel pixels from the background using vessel semantics; and multi‐source feature integration combines the outputs of the previous two modules to obtain the final A/V classification results.

## 3 Proposed Method

**FIGURE 3:**
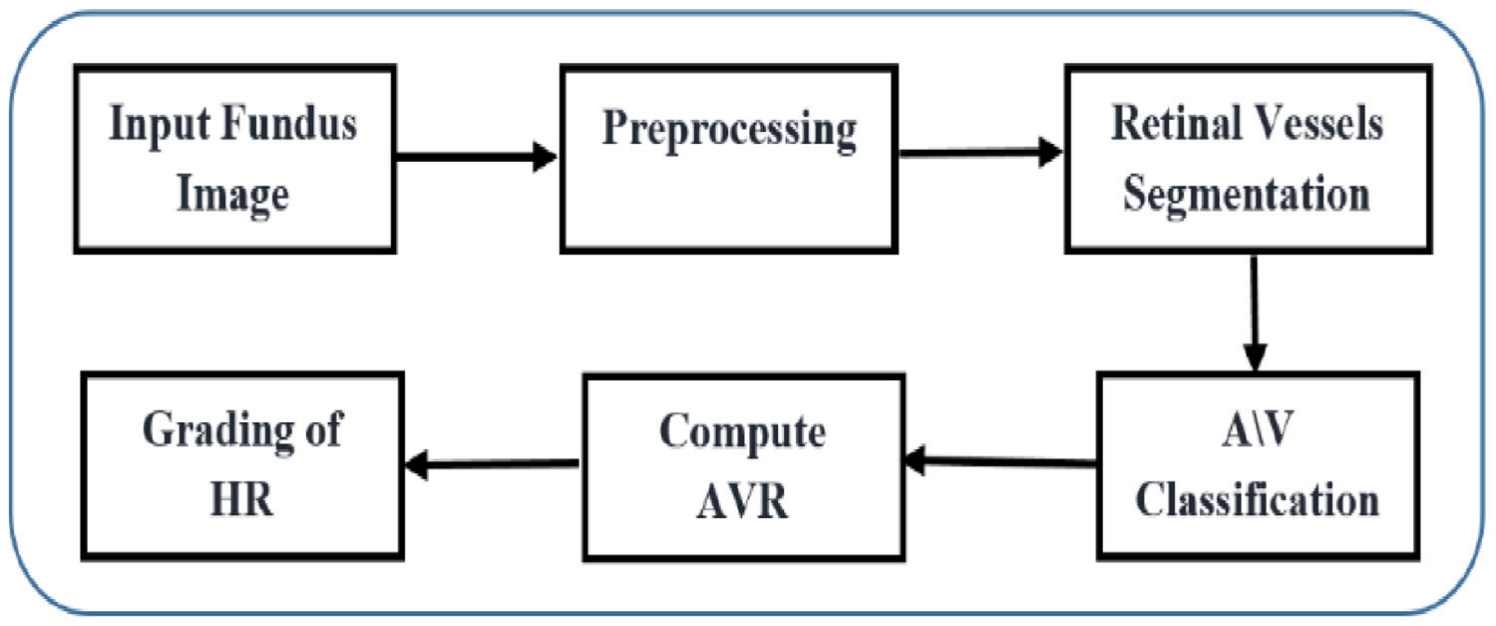
The structural organisation of the proposed technique.

The proposed method's contribution is its capability to distinguish between A/V in vessels, which is a crucial factor in detecting AVR and diagnosing HR based on retinal images. The proposed technique is presented in Figure 3.

The retinal vessel classification process starts with the input dataset and then the training of the Res‐UNet model involves extracting image features from the segmented vessels and subsequently utilising both the Res‐UNet features and the segmented vessels to create a graph representation that incorporates isolated nodes for pixels that do not belong to blood vessels. The graph is then classified using a GCN to extract its topological features. Finally, the AVR values are computed and the image is diagnosed as normal or HR. The model is presented in Figure 3.

The proposed approach follows a structured sequence of analytical steps that work together to extract clinically meaningful vascular information from retinal fundus images. The workflow begins by preparing the image through a set of preprocessing operations aimed at enhancing vessel visibility, reducing noise and correcting illumination variations. Once the vascular structures become clearer, the retinal vessels are segmented to obtain a detailed map of the vascular network. This segmentation output forms the basis for the next stage, where the vessel tree is translated into a graph representation that reflects how vessel points are naturally connected within the retina. Converting the vascular network into a graph allows the system to examine both the appearance of each vessel segment and its structural relationship to surrounding segments, which supports a reliable distinction between A/V.

After separating arteries from veins, quantitative measurements such as CRAE, CRVE and AVR are computed to characterise the vascular changes associated with hypertensive retinopathy. These measurements provide objective indicators of vessel narrowing or dilation and contribute directly to the final diagnostic decision. Figure 3 summarises the complete workflow, illustrating how each stage feeds into the next to produce a consistent and interpretable assessment of hypertensive retinopathy.

### 3.1 Datasets

**FIGURE 4:**
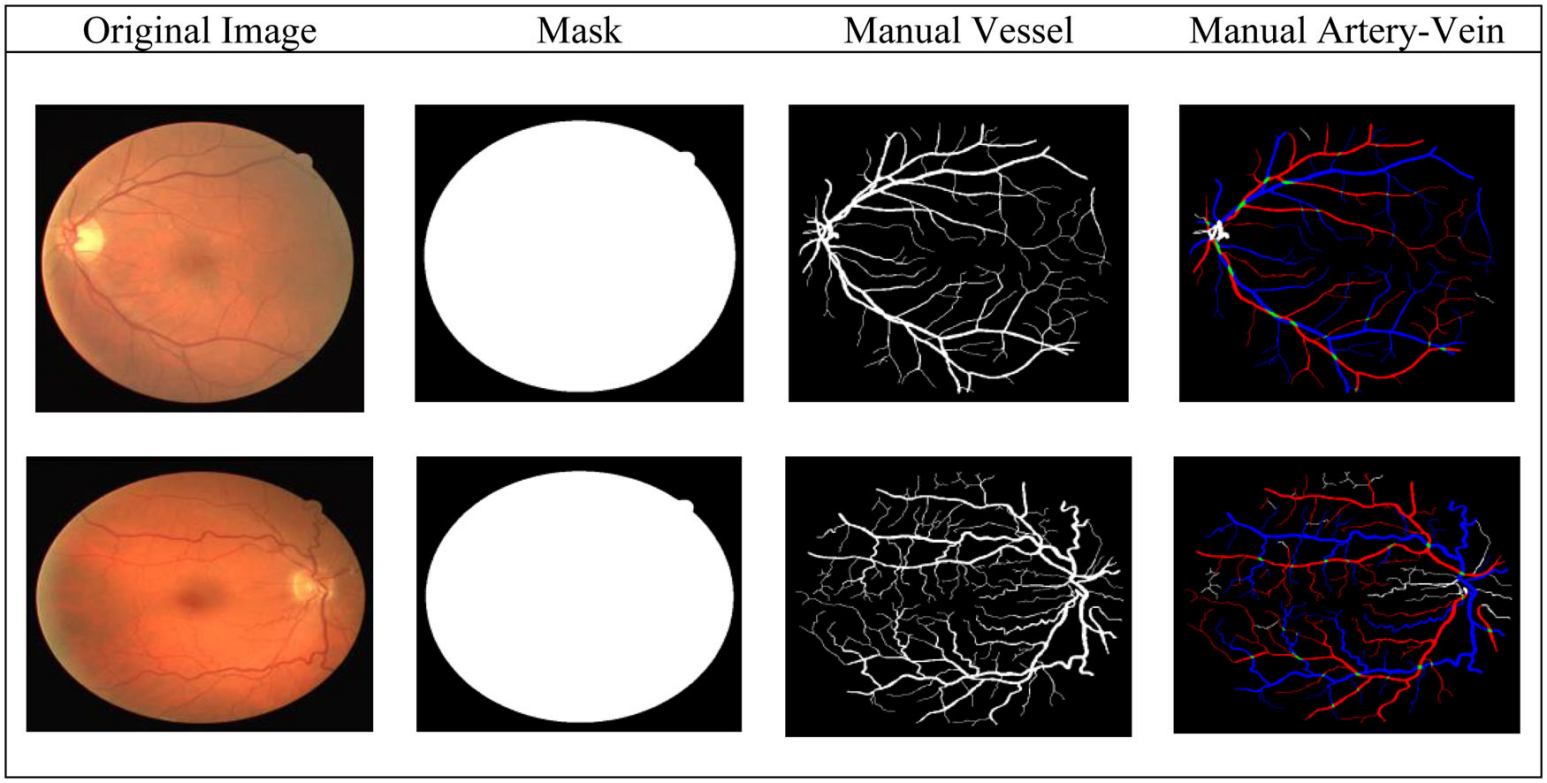
AV‐DRIVE datasets' sample images, mask, vessel and artery‐vein labels. Pixels labelled red and blue correspond to arteries and veins. In the AV‐DRIVE data set, undefined vessels are indicated with a green label.

The DRIVE dataset is openly available to the public and has become a popular standard for retinal vascular segmentation research. The dataset is composed of 40 fundus images, with half of them 20 allocated for training and the remaining 20 for testing purposes. Each image has a resolution of 584 × 565 and includes ground truth labels for vessel extraction, along with binary masks to determine the field of view. A 45° field of view non‐mydriatic 3 CCD camera was used to capture the images. To obtain a filtered red, green and blue colour range for flight, this type of camera features three separate charge‐coupled integrated circuits. The DRIVE dataset was employed to evaluate the A/V classification accuracy as well. Moreover, the label for AV in a previous study was obtained using the second manual observer's binary vessel segmentation ground truth. Two gold standards were used for A/V classification. The labels and images have a resolution of 584 × 565 pixels. It has been used for several tasks related to retinal image processing, such as segmentation, vascular registration and the advancement of retinal disease. A benchmark dataset for assessing artery‐vein classification is 25 AV‐DRIVE, which includes classification labels for both large and capillary arteries. The average disc occupancy on the DRIVE image, in contrast to the LEI‐CENTRAL image, is only around 2.18% of the total retinal content on an image. The majority of the optic disc vessel pixels in the optic disc centre were labelled as undefined rather than as an artery or vein. The ground truth masks for AV‐DRIVE are accessible to all. Qureshi et al. [30] report that three observers (two computer vision specialists and one ophthalmologist) performed artery‐vein labelling. By exchanging and debating contradictory labels among themselves, 25 observers updated their conflicting labels for the few vessels. They established several assumptions on the properties of the retinal vascular system, such as the fact that the arteries are often thinner and brighter than the nearby veins and that the vessels crossing one another must belong to different classes. A retinal image, together with its binary vascular segmentation and artery‐vein labelling from AV‐DRIVE datasets, is displayed in Figure 4.

### 3.2 Preprocessing

Retinal fundus images commonly suffer from several quality limitations that arise during acquisition, such as non‐uniform illumination, variable brightness, camera‐induced noise and differences in pupil dilation. These inconsistencies can significantly reduce the visibility of the retinal vasculature and negatively affect the performance of vessel segmentation and subsequent artery–vein classification. Therefore, a dedicated preprocessing stage was incorporated to standardise the visual appearance of the images and enhance the contrast between the vessels and the background before applying the segmentation model. The preprocessing pipeline begins with extracting the green channel from the RGB fundus image. This channel is known to provide the strongest natural contrast between blood vessels and surrounding retinal tissue due to the absorption characteristics of haemoglobin. As a result, major vascular structures as well as smaller capillary branches appear more distinguishable in the green channel compared to the red or blue components. To further improve vessel visibility, contrast‐limited adaptive histogram equalisation (CLAHE) was applied. CLAHE enhances local contrast by redistributing pixel intensities within small, contextual regions while preventing over‐amplification of noise through its clipping mechanism. This method is particularly effective for fundus imaging, where the brightness often varies across the field of view. By applying CLAHE, the darker regions surrounding thin arteries become clearer and the contrast between arteries, veins and background tissue is substantially increased. This enhancement is crucial for supporting downstream stages, especially the segmentation of faint vessel branches that would otherwise be difficult to detect. In addition to contrast enhancement, a mild noise‐suppression filter was applied to reduce sensor noise without compromising fine vascular details. This step ensures that the segmentation model receives a smoother and more uniform input, improving its ability to learn vessel boundaries consistently across images from different datasets. The combined effect of these preprocessing operations results in a clearer and more standardised representation of the retinal vasculature, enabling the segmentation network and the A/V classification module to achieve more reliable and robust performance.

Fundus images, due to their tiny pupil size and potential for over‐ or under‐exposure during capture, can suffer from nonuniform lighting difficulties that appear as low‐frequency artefacts across the image. As CNNs use small image patches for training inputs, they are unable to address these lighting errors without assistance. To prepare the datasets for training, they undergo preprocessing that includes various transformations to remove unwanted variations and simplify the training process. Due to the crooked shape of the retina, which causes large brightness changes in the image, these modifications are required. To enhance retinal image quality and make training easier, preprocessing steps are taken. These steps include increasing the size of the data set by applying the data augmentation technique, then extracting the green channel for increased contrast and using CLAHE to increase the difference between the lightest and darkest areas of an image.

#### 3.2.1 Data Augmentations

To increase the diversity of the training samples and reduce the risk of overfitting, several data augmentation techniques were applied to the original images. Fundus datasets are typically limited in size and retinal images often exhibit variations in patient positioning and camera orientation. Therefore, augmentation plays an essential role in enabling the model to generalise well across different imaging conditions. Among the applied augmentation methods, image rotation was particularly important due to its relevance to real‐world fundus acquisition. During retinal imaging, slight head movements, camera tilt and unintended patient repositioning can naturally lead to changes in image orientation. To simulate these conditions, the images were rotated using a set of predefined angles that reflect both subtle and more pronounced orientation shifts. Specifically, small angles (± 20°) were used to reproduce minor variations commonly seen during clinical capture, while moderate angles (± 40° and ± 60°) represent more noticeable yet realistic deviations. In addition, rotations of 90°, 180° and 270° were included to ensure that the model remains invariant to major orientation changes and does not become sensitive to a specific viewpoint. By incorporating this range of rotation angles, the augmentation process exposes the model to multiple plausible orientations of the retinal structures, improving its robustness during both segmentation and A/V classification. Additional augmentation steps, such as horizontal flipping, brightness adjustment and slight scaling, were also applied to further enrich the training set and help the model adapt to natural variations in illumination and retinal appearance. Overall, the augmentation strategy significantly enhances the model's ability to generalise to unseen images and reduces dependency on the original dataset's limited size.

**FIGURE 5:**
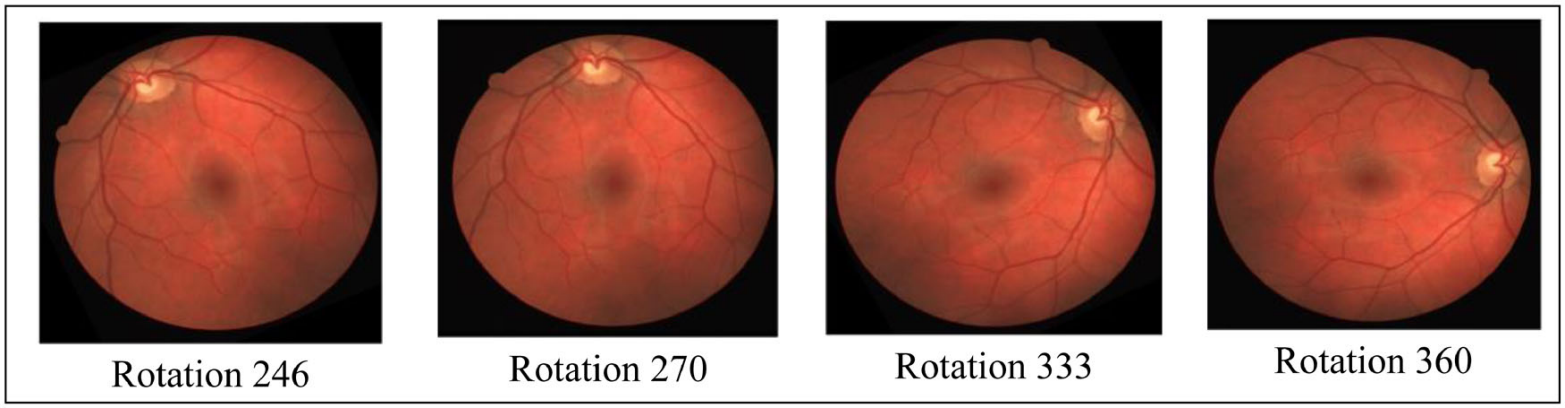
Sample results of the data augmentation process.

Is a method of making modified copies of a dataset using existing data to artificially increase the training set. To enlarge the dataset, it involves rotating images at a specific angle. This gives models access to a wider variety of training data, which enhances their performance. Some results of the data augmentation process are shown in Figure 5.

#### 3.2.2 Green Channel Extraction

**FIGURE 6:**
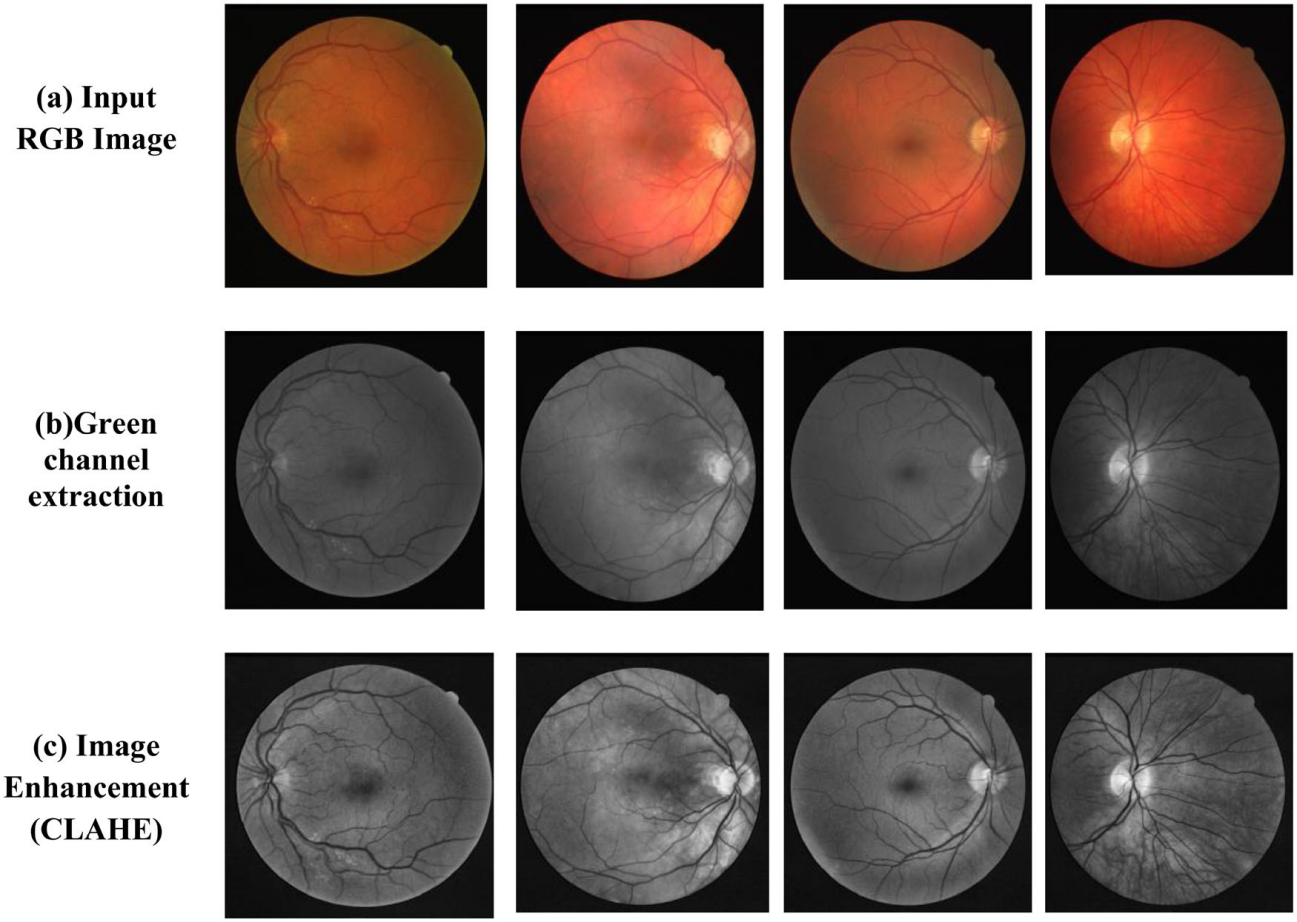
Sample result of image pre‐processing.

Green channel extraction is the technique of improving the image by selecting the green channel. Because of its great contrast and intensity in the retinal blood vessels, the green channel is one component of the RGB channels [31]. Equation (1) is the mathematical equation that was utilised to determine the green channel.

(1) $$G_{ch}=\frac{G}{R+G+B}$$

where $G_{ch}$ is the green channel, ***R*** is the red component, ***G*** is the green component and ***B*** is the blue component. The Green channel extraction result is displayed in Figure 6b.

#### 3.2.3 Image Enhancement (CLAHE)

This technique might make the enhanced image more resistant to changes in local brightness and lessen the effect of fluctuating image brightness on classification results. After equalisation, the upgraded image will have a more dynamic range and contrast in its grey level of pixels. The image's pixels will be evenly spaced and occupy as many grey levels as possible. Equation (2) demonstrates the implementation of the histogram equalisation process.

(2) $$\;G_{f}=\frac{G_{\max}}{N_{0}}\;\sum_{i\;=0}^{G_{A}}\left(P_{i}\right)\;\left(\;G_{A}=0,1,2,\;\text{…},\;L-1\right)$$

In the equation, **Gf** is the grey value after conversion, ***G*max** is the maximum grey value in the image, ***N*0** is the total number of pixels, ***GA*** is the grey value before conversion, ***Pi*** is the number of pixels in level ***i*** grey scale and ***L*** is the largest pixel level in the pixels of the image. Histogram equalisation is widely used in image enhancement due to its ease of use, speed and efficiency [32]. The histogram equalisation result is displayed in Figure 6c.

### 3.3 Blood Vessels Segmentation

**FIGURE 7:**
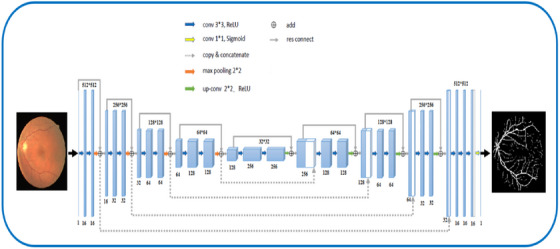
The proposed Res‐UNet model architecture.

The proposed model architecture shown in Figure 7 is designed to effectively identify blood vessels in the DRIVE‐AV data set. Due to their ability to learn and extract high‐level features from input images, Res‐UNet is often used for image analysis tasks, such as the segmentation of blood vessels in retinal images. An encoding branch (on the left) and a decoding branch (on the right) comprise the network structure. The encoding branch applies the basic residual block with two successive convolutional layers (same padding). A batch normalisation layer and a ReLU nonlinear layer come after each convolutional layer. After each basic residual block, a 2 × 2 max‐pooling operation is performed to perform down sampling. We double the number of feature channels at every round of down sampling. To restore the segmented output's space size, the decoding branch goes through the same number of up sampling operations. A 2 × 2 transposed convolution is used to accomplish each up sampling and the number of feature channels is reduced by half. Skip connections are used to copy features from the encoder to the decoder to aid in the decoding process. Basic residual blocks with two consecutive convolutional layers (same padding) are then used for feature extraction. Also, a batch normalisation layer and a ReLU nonlinear layer come after every convolutional layer. Instead of using the copy concatenation method, the encoded and decoded features are combined by summation. Concatenation is a natural approach to mix features from the encoder and the decoder, as it gives the up sampling process two input sources. However, there are two benefits to summarising characteristics using the additive technique [33]. First, since summing does not increase the number of feature maps, the training parameters in the following layer are decreased correspondingly. Next, it is possible to think of skip connections with summing as long‐range residual connections, which can help the model train more successfully. This network architecture makes use of some batch normalisation layers [34]. By standardising the inputs of the network's layers, BN layers help the neural network become more stable and expedite the training process. Additionally, under some conditions, the modest regularisation impact improves the model's output. During the encoding process, the convolutional kernels' receptive fields are multiplied in each down sampling step and the encoder continuously employs a stack of blocks made up of convolutional and max‐pooling layers to collect high‐level features. Continuous convolutional layers are used to rebuild the input data after the feature output from the preceding layer has been up sampled and merged with features from the skip connection during the decoding phase. The ultimate output size is identical to the original image size due to continuous up sampling. Furthermore, we have included residual structure, or short skip connections [35]. The residual structure facilitates network optimisation by lowering training calculations, improving model depth, accelerating convergence and improving accuracy without compromising model quality between low and high levels. The right mix of long and short connections complements semantic information at the high level and improves segmentation outlines at the low level, enabling the network to extract features at various levels and improve expressiveness. Finally, a sigmoid function and a 1 × 1 convolutional layer are used to project multi‐channel feature maps and provide the necessary segmentation outcomes.

**FIGURE 8:**
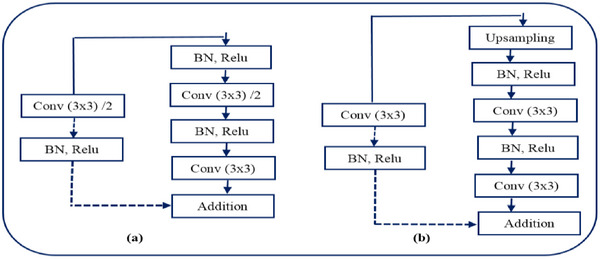
(a) Encoder block, (b) decoder block.

Figure 8a illustrates the structure of the encoder block. For our Res‐UNet network, residual connections and batch normalisation (BN) layers were added to normalise the top and lower sampling layers. The decoder block's structure (as seen in Figure 8b) is comparable to that of the encoder block, but all of its convolutional layers have a stride of 1. The residual connection is employed to prevent gradient vanishing in the deep network phase. The batch normalisation layer may significantly accelerate the rate at which the network converges and enhances the model's capacity for generalisation.

#### 3.3.1 Encoder‐Decoder Structure

An image will lose more semantic information when convolution and pooling are used often, even if each encoder and decoder block in the standard U‐Net layout has two convolution layers and a pooling layer. This process, known as gradient disappearance, may happen as the network gets deeper. The Resnet module takes the place of the conventional convolution module to prevent the gradient from fading and to hasten network convergence. Furthermore, to minimise the loss of image information, the conventional pooling method is replaced with the convolution operation with a step size of two. The dimensions of the feature map produced by the convolution operation are identical to those of the feature map produced by the conventional pooling technique. During model training, dropout is utilised to arbitrarily deactivate some neurons to avoid overfitting. Each convolutional layer in the paper uses linear correction units for feature extraction. Batch normalisation is used to normalise the activation value of hidden layer neurons. This can prevent the gradient from vanishing due to retinal noise and can improve the expression ability of the model. Relu can both speed up the network's convergence and significantly reduce its complexity. It uses the following formula:

(3) $$F\;\left(x\right)=\left\{\begin{array}{c} 0,\;x\leq 0\\ x,\;x>0 \end{array}\right.$$

where *F*(*x*) denotes the ReLU activation function.

#### 3.3.2 Feature Fusion Module

The retinal blood vessel image is more complicated and has significant variations in several regions, including the micro vessel and major blood vessel portions. We suggest a multiscale fusion technique using dilated convolution. To offer numerous effective fields of vision and identify segmented objects of varied sizes, the fusion approach primarily depends on variable dilation rates. The dilated convolution computation for two‐dimensional signals is represented mathematically as follows:

(4) $$y\;\left[i\right]=\sum_{k\;=1}^{K}x\left[i+r.k\right]\;w\left[k\right]\;$$

where *y*[*i*] denotes the output at position *i*,

*x*[⋅] is the input signal,

*w*[*k*] is the *k*‐th filter coefficient, *r* is the dilation rate, and *K* is the kernel size.

**FIGURE 9:**
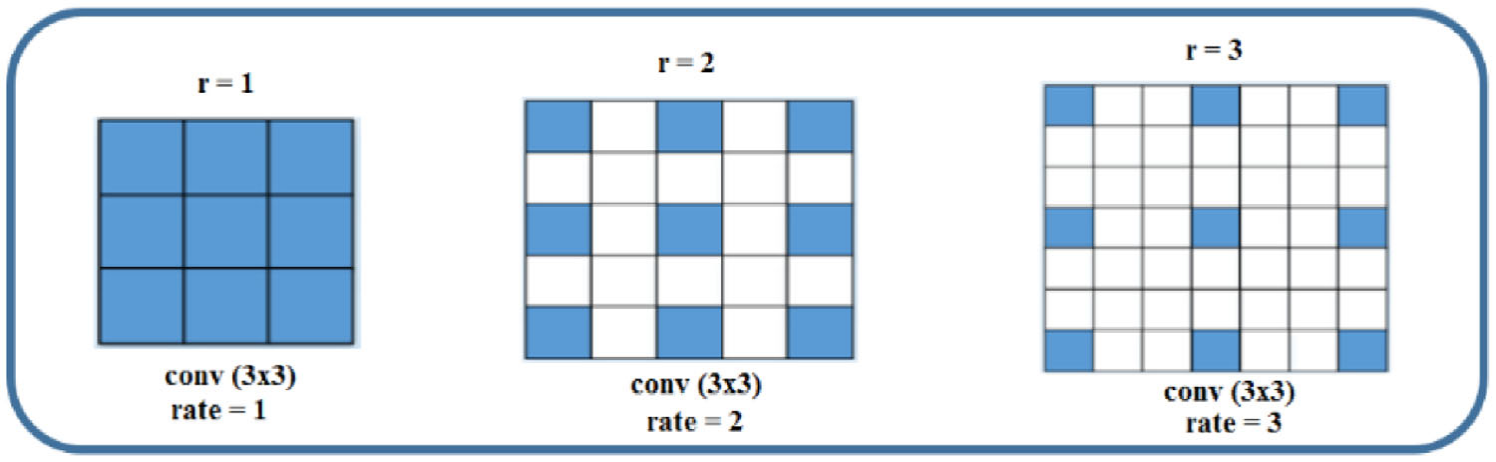
Schematic diagram of dilated convolution.

Among these, the stride of sampling the input signal is determined by ***r***, ***w*** and ***x***, which are the expansion rate, filter and input feature map, respectively. Convoluting input ***x*** with upsampled filters made by putting *r*‐1 zeros in between two successive filter values along each spatial dimension is the same as doing this. Figure 9 displays the schematic diagram of the dilated convolution.

The size of the receiving domain is the primary determinant of context semantic information. More context information can be used if the receiving domain can supply a greater amount of information. To extract visual features at different sizes, pooling techniques are typically performed to extend the receptive field. However, this also results in the loss of semantic information about the image. To address this limitation and efficiently extract features from images of varying sizes, we have implemented the previously described dilated convolution process. Dilated convolution is ideal for multi‐scale image segmentation applications because it can leverage the context information of the image and expand the receptive area freely without introducing extra parameters.

#### 3.3.3 The Skip Connection Scheme

Enhancing the accuracy and depth of deep CNNs can be achieved by using skip connections. We also incorporated skip connections, shown as the solid grey arrow in Figure 7, into our model after being inspired by this work. The following equation is used to build this skip connection technique for each convolutional block:

(5) $$y\;=\;F\left(x,\;\left\{w_{i}\right\}\right)+H\left(x\right),$$

where ***F*** is made up of two convolution operations, one max‐pooling operation and one up‐sampling operation. ***H*** can be either the same mapping or another convolution operation that maintains the input's feature dimensions.

#### 3.3.4 Robustness to Noise and Retinal Pathologies

Fundus images acquired in real‐world clinical environments often exhibit considerable variability due to camera noise, non‐uniform illumination, optical blur and the presence of additional retinal abnormalities such as microaneurysms, haemorrhages and exudates. These factors can distort vessel boundaries and alter local intensity distributions, posing challenges for automated vessel segmentation and subsequent artery/vein classification.

To mitigate these issues, the proposed framework incorporates several mechanisms that enhance robustness across heterogeneous imaging conditions. First, the preprocessing pipeline employs adaptive histogram equalisation to normalise illumination and enhance vessel‐to‐background contrast, while geometric normalisation minimises global structural variability between images. These steps reduce the influence of artefacts that commonly appear in retinal imaging.

Furthermore, the data augmentation strategy includes controlled rotations, random flips and mild intensity perturbations. Such augmentation encourages the model to learn vessel representations that are invariant to common distortions, thereby improving its generalisation capability across diverse imaging scenarios.

The graph‐based representation introduced in the GCN module further contributes to robustness. Unlike pixel‐intensity‐based methods, the graph structure encodes topological relationships between vessel segments, enabling the model to preserve connectivity even when parts of the vasculature are partially occluded by pathological lesions. By relying on the structural and relational properties of the vascular network rather than local pixel variations alone, the GCN component supports stable artery–vein differentiation under noisy or pathological conditions.

Overall, the integration of preprocessing normalisation, augmentation‐based variability modelling and graph‐driven topological reasoning enhances the system's resilience to noise and co‐existing retinal abnormalities, ensuring consistent A/V classification in practical clinical imaging environments.

After detailing the model's robustness to noise and pathological artefacts, we proceed to outline the loss functions employed during training to ensure stable convergence and accurate vessel classification.

#### 3.3.5 Loss Function

To train the suggested model, we select the binary cross‐entropy as the segmentation loss function. The loss function formula is constructed as follows:

(6) $$L\left(p,q\right)=-\frac{1}{n}\;\sum_{i}^{n}q_{i}\log\;p_{i}+\left(1-\;q_{i}\right)\log\left(1-\;p_{i}\right)$$

where ***p*** and ***q*** stand for the predicted pixel value and its matching ground truth and *n* is the number of pixels in each image. We employ adaptive moment estimation (Adam) as the model training process optimisation technique in the Res‐UNet network training process to enhance its training performance and produce better segmentation results. The Adam optimiser offers several benefits over typical optimisation algorithms, including adaptive learning rate adjustment, low memory consumption and great processing efficiency. It also has a natural annealing effect and can handle noisy samples more effectively.

### 3.4 Classification of Artery/Vein Using Graph Convolutional Network (GCN)

The retinal vascular tree has different visual characteristics that can be utilised to classify the A/V within it. Arteries are more visible and have smaller calibres compared to veins; however, diseases can change the calibre of vessels, resulting in an unreliable feature for A/V classification. A more dependable feature of arteries is their thicker walls, which create a shiny central reflex strip. Additionally, the vascular tree near the optic disc shows a pattern where veins and arteries rarely cross each other; they can bifurcate and come into contact with one another, enabling tracking and analysis of the vessel tree. This has been utilised in some methods to classify the vessels. The skeleton of the vessels served as the graph nodes in the first suggested application of graph convolution for retinal vessel classification. The vascular skeleton provided the graph edges, while Res‐UNet feature maps at the node positions provided the features for the graph node. Graph‐based methods create a visual representation of a vessel network by dividing it into several smaller subtrees, based on the connectivity of the vessels at branching and intersection points. These subtrees are then classified as either arterial or venous based on the classification of the vessel centrelines. In contrast, feature‐based methods use only the intensity information of individual pixels to differentiate between arterial and venous vessels. GCN is a particular kind of deep learning model that analyses graph topologies and uses the Res‐UNet technique to understand how nodes and edges are connected. Since many tissues or organs in medical images have structures comparable to graph structures, GCN is naturally used in medical image analysis. For instance, fundus images show blood vessels with a tree architecture. GCN performs well when displaying spatial data. Graph convolutional networks (GCNs) use weight parameters in each network layer to fundamentally handle cyclic mutual dependencies [36]. By propagating node information via edges, the spatial graph convolutional operation primarily focuses on aggregating and updating node representation [37]. Through aggregating information from neighbouring nodes and updating the centre node representation, the aggregation technique may directly improve the generalisation capability of handling varied structured networks. Initially, GCNs identify the intra‐ and inter‐regional connections among various tissue areas to derive multi‐scale spatial associations. Additionally, GCNs have a robust fusion capacity to manage heterogeneous cross‐modality imaging data. Since it can expand our knowledge of disease mechanisms beyond what single‐modality data can provide, the cross‐modality study is highly interesting. In conclusion, GCNs offer a means of understanding results by encapsulating the structural dynamics of complex graphs. The model results may be used to show the subgraph connectivity and node distribution obtained from the whole graph representation. When put together, GCNs show promise in analysing the vast quantity of graph‐level data necessary to improve our comprehension of medical imaging and guide clinical decision‐making. In exponential science, the term ‘graph’ in GCN refers to the topological graph where vertices and edges are utilised to find relevant correlations. GCN works directly on a graph, producing the node embedding vector based on the characteristics of the node's neighbourhood [38]. A graph is defined as *G* = (*V*, *E*), where the links between nodes are represented by *E* and the graph's vertex set is represented by *V* = {*Vi* |*i* = 1,…, *n*}. The definition of the feature matrix is *X* = [*x*1, *x*2,…, *xn*] ∈R *^n × m^*, where n is the number of nodes, *m* is the feature dimension and xi is the feature vector of the node v. The adjacency matrix of Graph G, *A* ∈ R *^n^* ^×^ *^n^*, indicates the adjacency connection between any two vertices; if nodes *vi* and *vj* are linked, then *aij* = 1, otherwise *aij* = 0. Every node is always linked to itself for consistency, thus *aii* = 1. *Dii* = Σ*_j_Aij*, where *D* is the degree matrix. The vertex's degree, or the number of components it links, is represented by the element on the diagonal.

**FIGURE 10:**
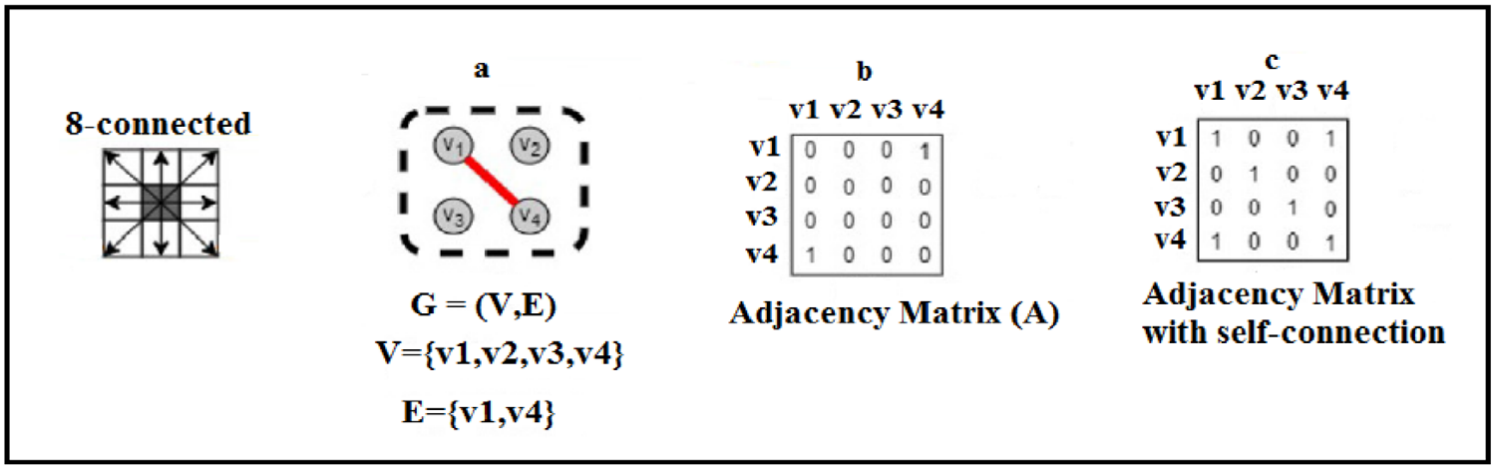
(a) simple example of an undirected graph, (b) the adjacency matrix (A), (c) the adjacency matrix with self‐connections($\hat{A\;}$).

Figure 10 displays a basic illustration of an undirected graph; the adjacency matrix (A) and the adjacency matrix with self‐connections ($\hat{A\;}$) of this graph are represented by (b, c).

**FIGURE 11:**
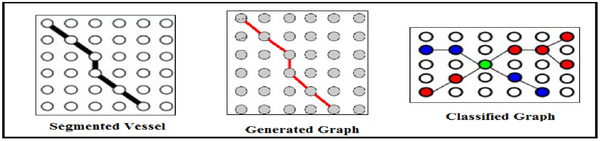
Generated graph for the segmented vessels.

The underlying retinal vascular structure, which the segmented vessels have captured, serves as a reference for the connection between the input image's pixels. Thus, we build our graph representation using the segmented vessels. To be more precise, *G* treats every pixel in the segmented vessel mask as a node. In *G*, an edge joins two neighbouring pixels if they are both assigned to the vessel class. Next, we investigate each pixel's 8‐connected neighbourhood (shown in Figure 11) for every node, or pixel, in our graph representation *G*. (*vi*, *vj*) ∈ *E* and the adjacency matrix *A* of *G* is updated if and only if both neighbouring *vi* and *vj* belong to the segmented vessels. *G* represents background pixels (non‐vessel pixels) as separate nodes.

Features representation, features transform and nonlinear activation are the three steps of operations in a graph convolution layer. Two components make up the GCN input: the adjacency matrix *A* ∈ R *^N × N^* and the feature matrix *X* ∈ R *^N × d^*. Nodes' features are denoted by *X*, whereas A represents the graph structure, *N* denotes the number of categories and d denotes the number of node features. Each GCN hidden layer can be defined as:

(7) $$\;H^{L+1}=f\left(\;H^{L},\;A\right)$$

The graph‐level outputs of the Lth layer are represented by *H^L^* ∈ R *^N × d^*. *H* ^0^ is the feature matrix, *X*, *A* is the adjacency matrix and *d* denotes the dimensionality of the node features. *f* (·) may be stated as follows using the propagation rule that was proposed in Kipf et al. [39]:

(8) $$f\;\left(\;H^{L},\;A\right)=\sigma \left(\hat{A}\;H^{L}\;W^{L}\right)$$

where $\hat{A}$∈ R *^N × N^* is the normalised form of correlation matrix *A*, W^L^ is the weight matrix for the *L*‐th neural network layer, Kipf et al. [35] calculated the weight matrix using Chebyshev polynomials based on the spectral graph convolution: $W=D^{-\frac{1}{2}}\;A\;D^{-\frac{1}{2}}$, where *D* is the diagonal degree matrix of *A*, $\;W_{ij}=\frac{a_{ij}}{\sqrt{d_{i}.d_{j}}}$. σ(·) is a non‐linear activation function. In this experiment, we used ReLU as the activation function. For the last layer, the output of GCN is *T* ∈ R *^N × D^*, *T*′∈R *^D × N^* is obtained by *T* transpose, Dim is the feature dimension of the final node. Finally, a softmax classifier is used in the final layer of a GCN to predict the labels for node classification, much like a conventional MLP. The predicted scores (*y*_predicted_) are selected by applying a classifier to the image features using the label features learned by the GCN. This is achieved by a linear transformation of *T*' and the label features and the predicted scores represent the probability of each node being classified into various categories, we can obtain the predicted scores ypred ∈ R *^N^* as:

(9) $$\;y_{\mathrm{pred}}=F\;\overset{`}{T}$$

**FIGURE 12:**
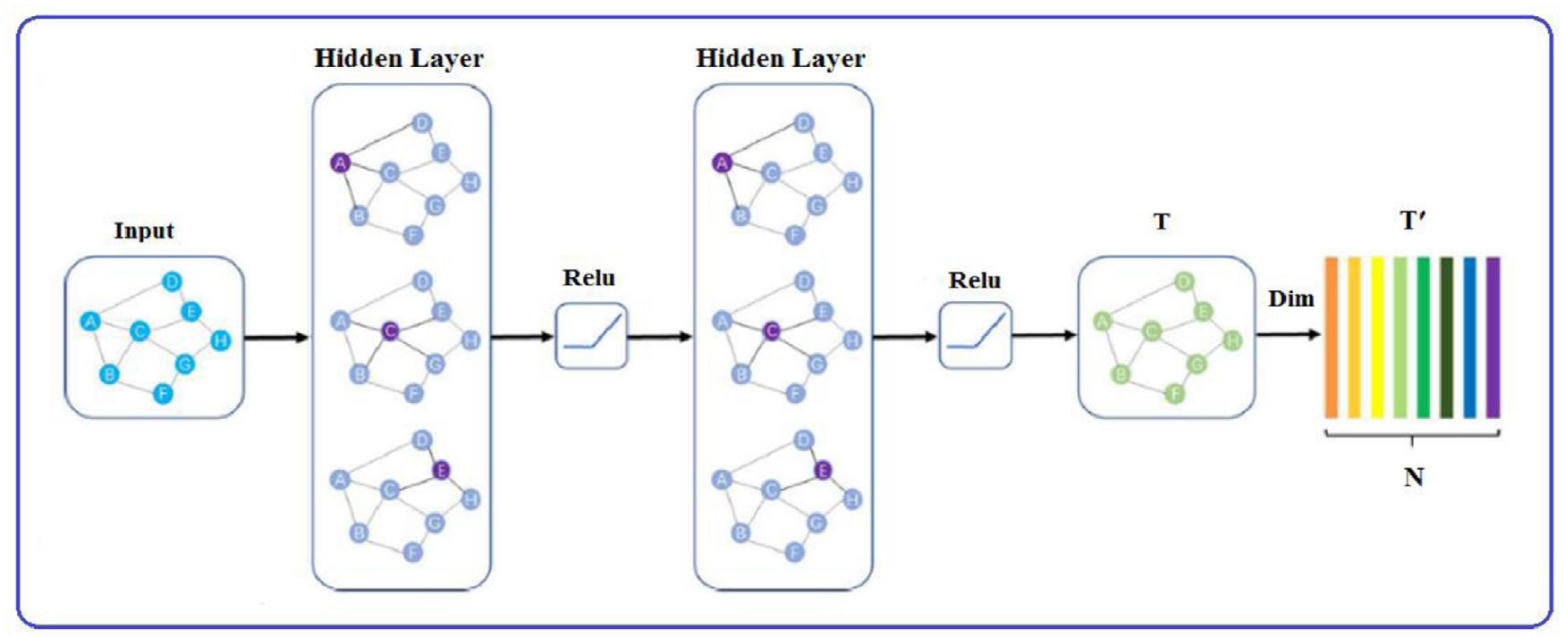
The GCN architecture diagram.

The GCN architecture used in this article is shown in Figure 12.

#### 3.4.1 Loss Function

We employ cross‐entropy (CE) loss and dice loss in the retinal vascular classification. Dice loss may automatically apply weights to samples of different classes to achieve the proper balance between foreground and background pixels, hence alleviating the problem of sample foreground and background imbalance. Dice loss calculates the similarity between two binary inputs. The definition of the dice loss is as follows:

(10) $$D\;\left(g,\hat{g}\right)=1-\;\frac{2\sum_{i=1}^{N}g_{i}\hat{g_{i}}+\varepsilon }{\sum_{i=1}^{N}{g_{i}}^{2}+\sum_{i+1}^{N}{\hat{g_{i}}}^{2}}$$

where ​ $g_{i}$ represents the ground‐truth label of pixel i, $\hat{g_{i}}$ is the predicted probability for that pixel, *N* is the total number of pixels and ε is a small constant added for numerical stability.

In classification and segmentation, CE loss is a widely used loss function that has the following definition:

(11) $$CE\;\left(b,\hat{b}\right)=-\frac{1}{N}\sum_{i\;=1}^{N}b_{i}\;\log\left(\hat{b_{i}}\right)+\left(1-b_{i}\right)\log\left(1-\left(\hat{b_{i}}\right)\right)\;$$

where $b_{i}$∈{0,1} denotes the ground‐truth label of pixel *i*, $\hat{b_{i}}$∈ [0,1] is the predicted probability for that pixel, *N* is the total number of pixels and CE ($b,\hat{b}$) denotes the binary cross‐entropy loss.

### 3.5 Computation of AVR

According to the medical literature, evaluating changes in the AVR and vascular tortuosity can aid in the identification of HR. A healthy patient's AVR is approximately 0.667, but an HR patient's AVR is between 0.2 and 0.5 [39]. The retinal vasculature can be used to measure these pathologies via a fundus image.

**FIGURE 13:**
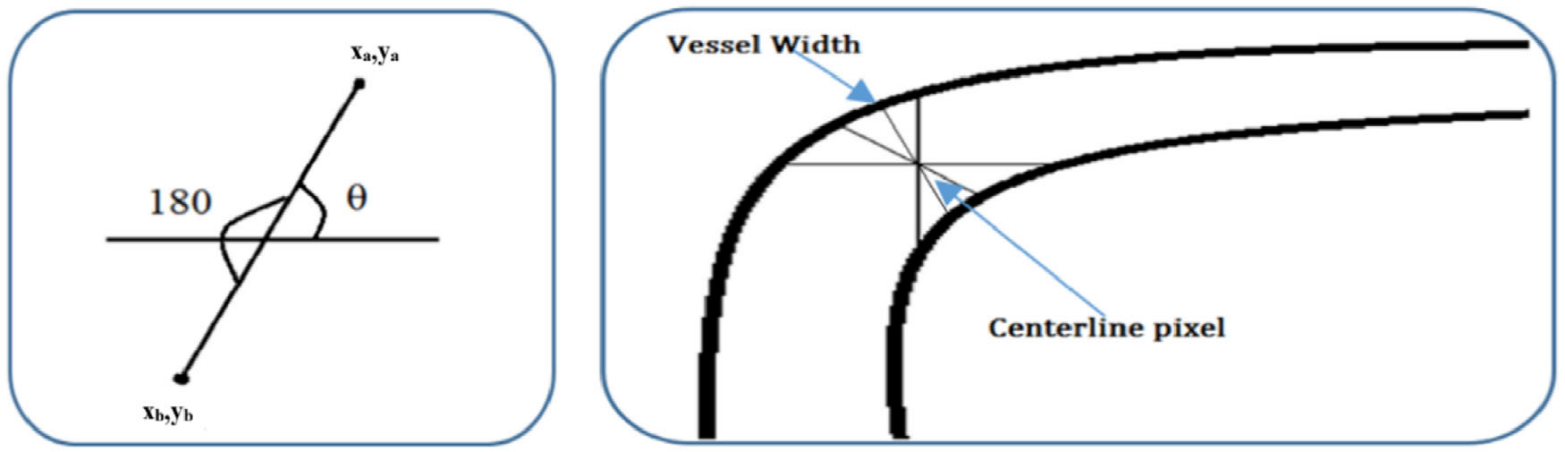
(a)Determining the mirror pixel. (b)The width of the vessel.

Veins have lower average pixel values than arteries, making arteries appear brighter when contrasting the two types of blood vessels. As a result, when compared to veins, the mean of their pixel values is more obvious. The width of the vessels is determined by finding the smallest distance between each point on one side of the vessel and the corresponding point on the other side. As shown in Figure 13, the centreline pixel is then roughly located if the vessel's edge is discovered. If an edge pixel's grey value (x_a_,y_a_) is 255, the matching edge pixel value on the other edge (x_b_,y_b_) may be obtained by changing the value of *θ* = *θ* + 180. By calculating the Euclidean distance between the edge pixels and taking into account an imaginary line that passes through the centre line pixel, the width of the cross‐section can be determined. The vascular width is determined by finding the shortest path between the vein and artery boundaries. The Euclidean distance is given as:

(12) $$E\;=\sqrt{{\left(x_{a}-\;x_{b}\right)}^{2}+{\left(y_{a}-\;y_{b}\right)}^{2}\;}\;$$

where (*x_a_*, *y_a_*) and (*x_b_*, *y_b_*) represent the coordinates of the two edge pixels located on opposite sides of the vessel cross‐section and **E** denotes the Euclidean distance used to estimate the vessel width.

The geometric interpretation of vessel width estimation and centreline‐based measurement is illustrated in Figure 13.

To compute the central retinal artery equivalent (CRAE) and central retinal vein equivalent (CRVE), the diameters of the largest arteriolar and venular branches are first measured within a predefined annular region around the optic disc. Let $D_{a1}$​ and $D_{a2}$ denote the calibrated diameters of two selected arterial branches and let $D_{v1}$ and $D_{v2}$ denote the corresponding venous diameters. These diameters are obtained directly from the segmented vessel map by locating the vessel centreline and measuring the Euclidean distance between the two edge pixels across the vessel cross‐section, as described in Equation (12).

Once the branch diameters are extracted, the Parr–Hubbard formulas are applied to combine the paired diameters into a single representative value for A/V [40]. CRAE and CRVE are computed using Equations (13) and (14), where the coefficients are taken from the original epidemiological models and remain constant for all images.

(13) $$\mathrm{CRAE}=\sqrt{{D_{a1}}^{2}+1.01{D_{a2}}^{2}-0.22D_{a1}D_{a2}-10.73}\;$$

(14) $$\mathrm{CRVE}=\sqrt{1.72{D_{v1}}^{2}+0.91{D_{v2}}^{2}+450.02}\;$$

From a clinical perspective, CRAE and CRVE provide important insight into the structural condition of the retinal microvasculature. A reduction in CRAE reflects arteriolar narrowing, an early hallmark of hypertensive microvascular damage caused by increased vascular resistance and arteriolar wall thickening. Conversely, an elevation in CRVE indicates venous dilation, which is associated with impaired venous drainage and elevated retinal venous pressure. To summarise these complementary changes, the arteriovenous ratio (AVR) is defined as follows:

(15) $$AVR=\frac{CRAE}{CRVE}\;$$

Lower AVR values are typically observed in hypertensive retinopathy due to the simultaneous narrowing of arteries and widening of veins. Therefore, AVR serves as a clinically meaningful indicator that correlates with the severity of hypertensive retinal changes and assists in automated HR detection and grading.

### 3.6 Grading of HR

The AVR value, along with the signs and symptoms of HR, can be used to classify HR into mild, severe and malignant phases. In the mild stage, symptoms such as arteriolar narrowing, arteriovenous nipping, copper wire appearance, silver wiring and increased tortuosity of the blood vessels may be present but are often ignored or difficult for ophthalmologists to detect. In the moderate stage, more obvious signs of retinopathy, such as cotton wool spots, hard exudates and flame‐shaped haemorrhages, can be easily observed by ophthalmologists. The final and most severe stage of HR is the malignant stage, which includes symptoms such as optic disc swelling, papilledema and all of the symptoms from the previous stages. In our work, we have classified the images as either having moderate HR or normal images. The HR grade is shown in Table 2.

**TABLE 2:** Evaluation of HR.

AVR	Signs and indications	HR grade
0.667–0.75	None	Normal retina
0.5	Arteriolar narrowing, arteriovenous nipping, copper wire appearance, silver wiring.	Mild
0.25	cotton wool spots, hard exudates and flame‐shaped haemorrhages.	Moderate
< 0.2	optic disc swelling, papilledema and all of the symptoms from the previous stages.	Malignant

## 4 Experiments and Results

### 4.1 Determine of Parameters

The system suggested in this study was created utilising the Python programming language and Keras framework, which utilises TensorFlow as its underlying technology. The system evaluations were performed on a GPU resource, specifically an NVIDIA Tesla K20 GPU with 8 GB RAM. Hyperparameters are variables in deep learning networks that are either pre‐selected by a computer designer or tuned using optimisation techniques, including random search, grid search, or gradient‐based optimisation. However, manual hyperparameter tuning is still widely used due to its speed. To update the model parameters for successful image detection, the stochastic gradient descent algorithm is utilised to minimise the cross‐entropy loss. The parameters of the max pooling layer, including the number of filters, are also crucial in determining the model's performance.

### 4.2 Performance Analysis

The assessment of the proposed methods' performance has been computed and analysed using various evaluation metrics. The evaluation parameters are described as:

‐ Accuracy: the degree of agreement between the result and the ground truths. The accuracy of the suggested algorithm has increased, suggesting an improved result. It is calculated using the formula:
(16) Accuracy=(TP+TN)∕(TP+FP+FN+TN)
‐ Sensitivity (recall): is a metric that indicates the ability of the proposed method to accurately recognise the correct pixels. It is typically calculated using a formula.
(17) Sensitivity=TP∕(TP+FN)
‐ Specificity: is a metric that evaluates the proposed method's precision in identifying pixels beyond the region of interest. It is calculated as follows.
(18) Specificity=TN∕(TN+FP)
‐ Precision call: Precision demonstrates that the percentage of the actual true positive test samples is flattering for each test sample that the model has recognised. Precision is calculated using formula (22):
(19) Precision=TP∕(TP+FP)
‐ F1 Score: it's used to display the harmonic mean of the Precision and Recall numbers. As shown in Equation (12).
(20) F1Score=2×(precision×recall)∕(precision+recall)
‐ The Matthews correlation coefficient (MCC): is often used to assess how well two different‐sized categories perform in binary classifications. As a result, the MCC value can be used to determine the vessel segmentation algorithm's ideal setting. MCC is described as:
(21) $$\mathrm{MCC}=\frac{\left(TP\;\times \;TN\right)\;-\;\left(FP\;\times \;FN\right)}{\sqrt{\left(\left(TP+FP\right)\left(TP+FN\right)\left(TN+FP\right)\left(TN+FN\right)\right)}}\;$$
‐ The area under the ROC curve (AUC): can be used to evaluate the model's performance. A perfect result is indicated by an AUC value of 1.

### 4.3 Results and Discussion

To assess our model, we split the outcomes of each pixel comparison into true positive (TP), false positive (FP), false negative (FN) and true negative (TN) categories after comparing the results with the relevant ground truth. The values for these categories are shown in Table 3. The model's performance is then assessed using the following metrics: accuracy (ACC), sensitivity (SEN), specificity (SP) and F1‐score, precision and correlation coefficient.

**TABLE 3:** Predicted outcomes values of the confusion matrix.

Model	TP	FP	FN	TN
**Res‐UNet**	974	26	45	955
**GCN**	977	23	43	957

The proposed technique on the DRIVE‐AV datasets. The results of the performance analysis of our proposed method for the segmentation of blood vessels and classification of AV on the datasets are shown in Table 4. The proposed technique achieved an accuracy of 96.45% for blood vessel segmentation and 96.7 % A/V classification.

**TABLE 4:** Average performance of the Res‐UNet models.

Model	Accuracy (%)	Sensitivity (%)	Specificity (%)	F1‐score (%)	Precision (%)	Correlation coefficient (%)
**Blood vessel segmentation** **(Res‐UNet)**	96.45	95.58	97.35	96.48	79.39	92.92
**A/V classification** **(GCN)**	96.7	95.78	97.65	96.73	97.7	93.42

**FIGURE 14:**
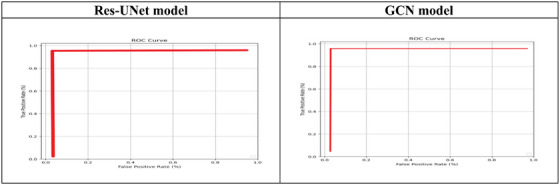
ROC curves for the proposed method on DRIVE‐AV datasets.

A test's sensitivity and specificity can be visually represented with a receiver operating characteristic curve (ROC). To create a ROC curve, samples that are deemed true or false are computed based on the test. The TPR (sensitivity) is mapped against the FPR (1 ‐ specificity) for certain cut‐off values. The ideal point is one that sits over the shoulder of the curve because it minimises false positives and increases actual positives. The ROC for the proposed method is displayed in Figure 14.

**FIGURE 15:**
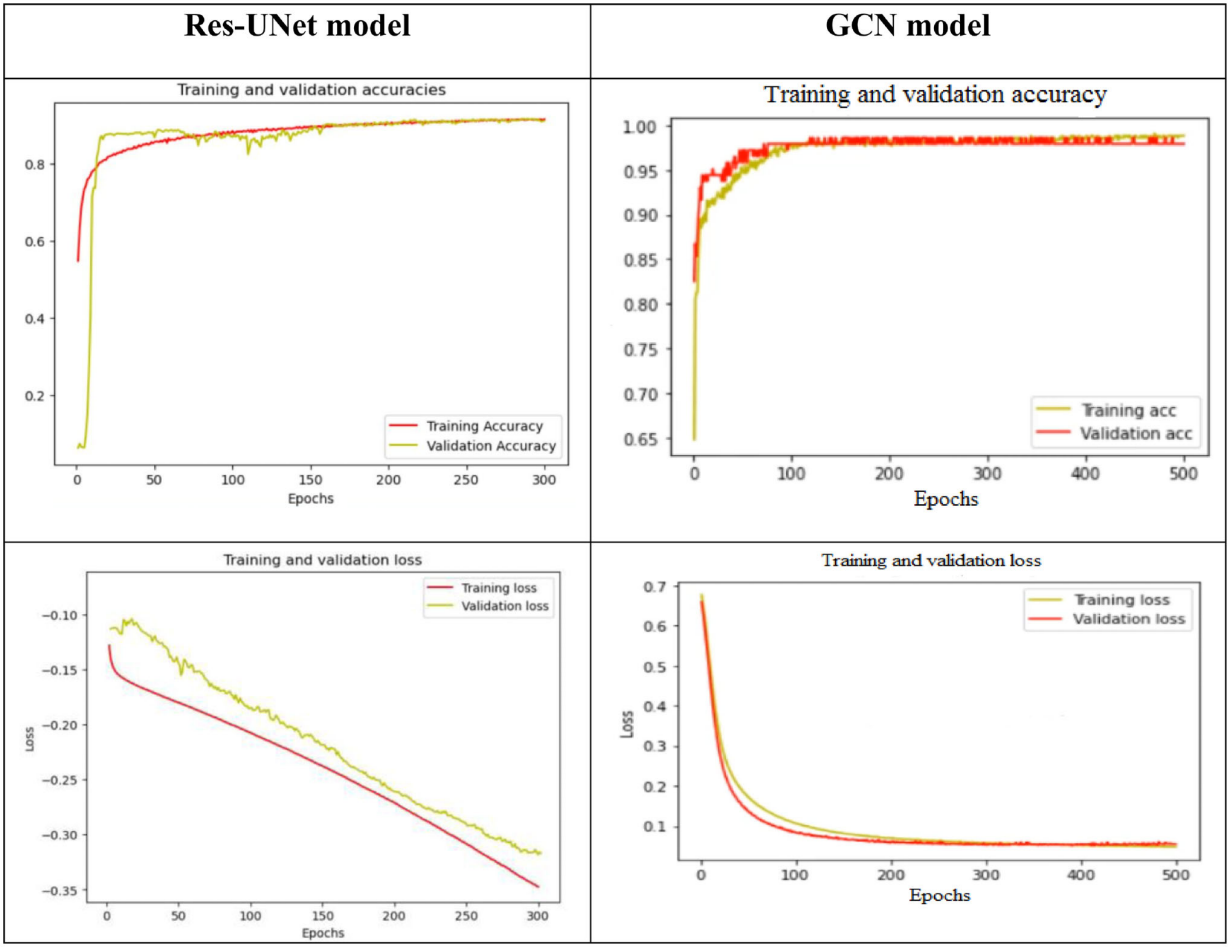
Validation accuracy and validation loss for the proposed model on the DRIVE‐AV datasets.

The network's requirements determine how many epochs are used. The dataset indicates that it is necessary to choose the quantity of filters and convolutional blocks. Saturation on the condensation image will occur with more blocks. Dropouts are arranged after each layer under that layer. To reduce the model's overfitting, dropouts are incorporated. It can reduce the reliance brought on by the fully connected layers by ignoring some visible and non‐visible layers. For each epoch, the model's losses throughout the training and validation phases are quite similar to one another. This shows that our model does not exhibit overfitting. With a test accuracy rate of 96.7% for the GCN model and 96.45% for the Res‐UNet model. The validation accuracy and validation loss for the suggested model using the DRIVE‐AV datasets are shown in Figure 15.

**FIGURE 16:**
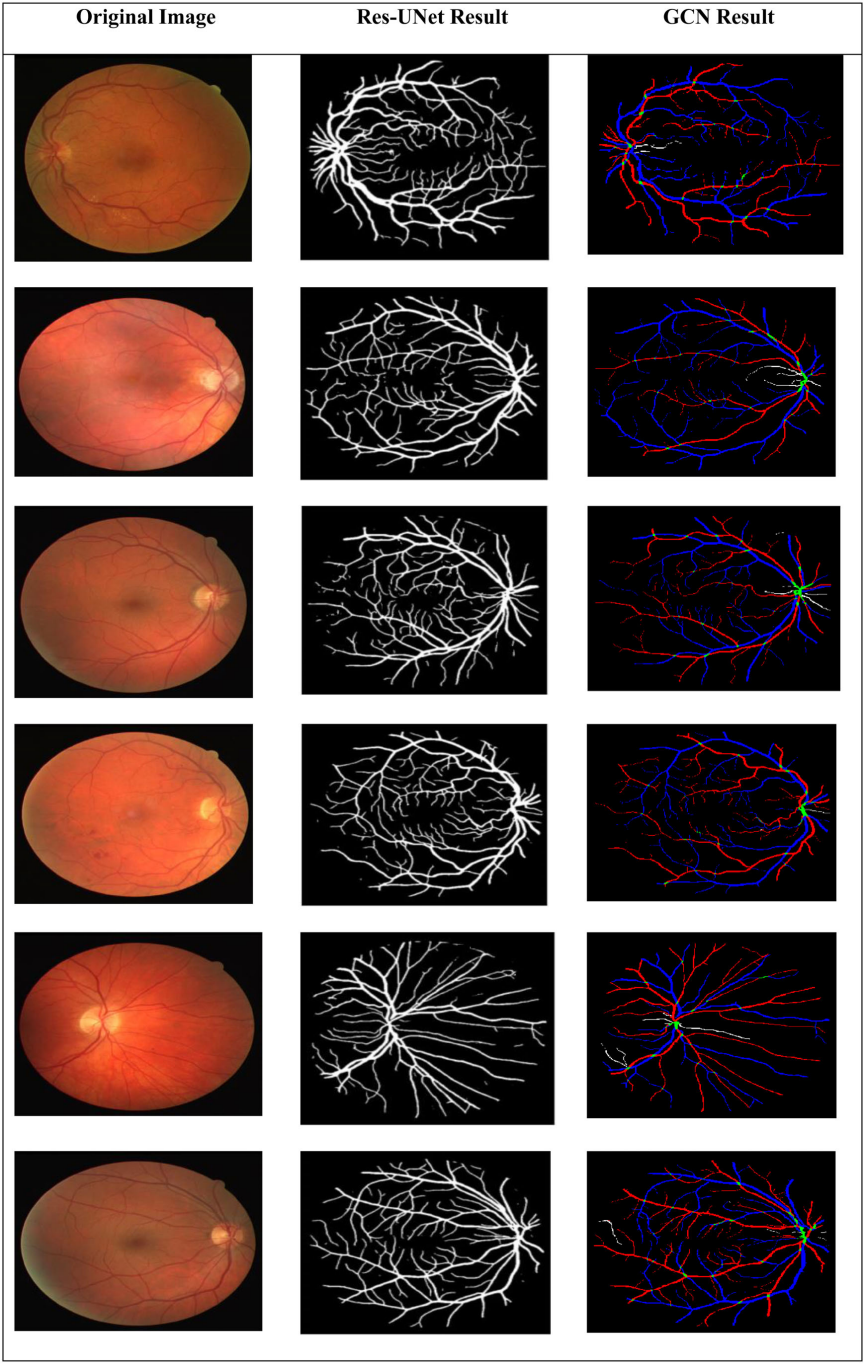
The results of retinal blood vessel segmentation and classification in the DRIVE‐AV dataset; original, ground truth and segmented image results, classified image results.

Figure 16 shows some segmentation and classification results generated by the suggested approach. The results demonstrate that our model can effectively handle challenges, such as low light levels and hard areas on the optic disk, among others. In addition, our model's results demonstrate improved vascular connectivity. It is also capable of precisely segmenting small, indistinct vessels and preserving the geometric connection of retinal vessels.

**FIGURE 17:**
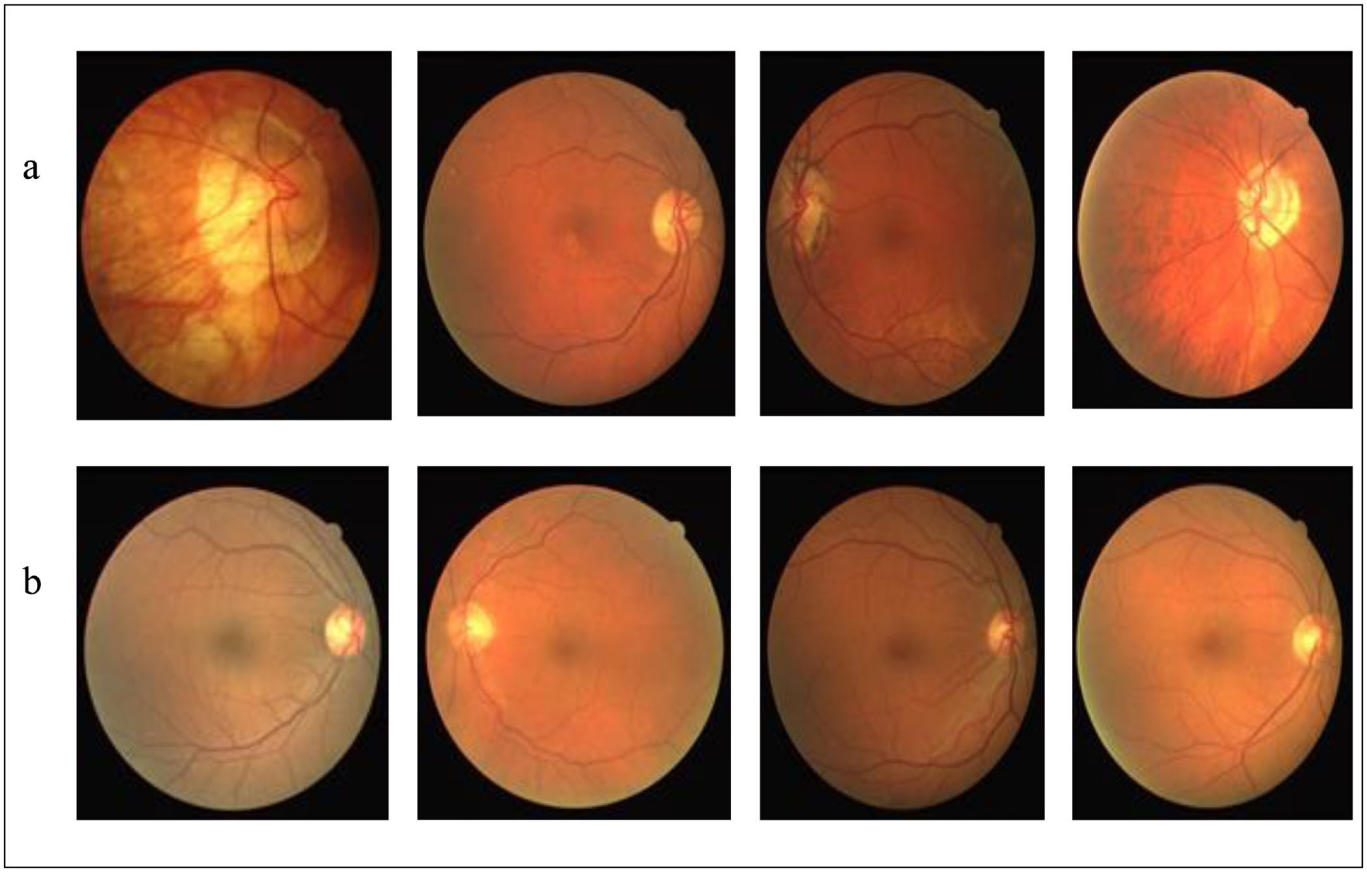
The proposed system generates two kinds of outputs. The first one (a) shows the images that are considered (HR) and the second type of output (b) indicates normal images.

The outcomes of the categorisation of HR grades using the datasets are visually represented in Figure 17. The figure includes two parts: Figure 17a displays the images that have been classified as HR and exhibit signs such as microaneurysms and haemorrhages. Figure 17b presents a normal image.

An overview of published research for hypertensive retinopathy detection is given in Table 5, along with details on the datasets, techniques and accuracy of each approach.

**TABLE 5:** Comparative analysis of different methods for detecting HR on the AV‐DRIVE dataset.

NO	Approaches	Year	Method	Performance
**1**	Xu et al. [18]	2017	kNN classifier	ACC: 92.3 %
**2**	Xu et al. [13]	2018	Fully convolutional network (FCN)	ACC: 90 %
**3**	Ma et al. [14]	2019	Neural network	ACC: 92.6 % SEN: 92.2 % SP: 90.3 %
**4**	Zhao et al. [19]	2019	Dominant sets clustering	ACC: 93.5 % SEN: 94.2 % SP: 92.7 %
**5**	Arsalan et al. [10]	2019	Convolutional neural network (CNN)	ACC: 80 % SEN: 78.5 % SP: 81.5 %
**6**	Srinidhi et al. [20]	2019	Depth‐first search (DFS), RF	ACC: 94.7 % SEN: 96.6 % SP: 92.9 %
**7**	Noh et al. [41]	2020	Hierarchical vessel graph network	ACC: 92.6 % SEN: 93 % SP: 92.2 %
**8**	Kang et al. [21]	2020	AVNet	ACC: 90.81% SEN: 88.63% SP: 92.72 %
**9**	Li et al. [22]	2020	SeqNet	ACC: 91.90 % SEN: 90% SP: 84 %
**10**	Abbas et al. [26]	2021	Convolutional neural network (CNN)	ACC:92.9 % SEN: 90.5 % SP: 91.5%
**11**	Chowdhury et al. [23]	2022	MSGANet‐RAV	ACC: 93.15 % SEN: 92.19 % SP: 94.13 %
**12**	Yi et al. [27]	2023	U‐Net	ACC: 86.56 % SEN: 87.42 % SP: 86.32 %
**13**	Kadry et al. [42]	2024	Res‐UNet	ACC: 89.8%
**14**	Sheejakumari et al. [43]	2025	MM‐Net	ACC: 89.2%
**15**	**Proposed Method**	**2025**	**Res‐UNet** **GCN**	**ACC: 96.3%** **ACC: 96.7%**

Although certain studies, such as Arsalan et al. (2019), report slightly higher accuracy values (approximately 97%), it is important to note that their performance is achieved using a substantially larger training pool constructed from two independent datasets, combined with testing on a third external dataset. This multi‐dataset training paradigm naturally improves model generalisability but relies on the availability of consistent artery/vein annotations across datasets — an aspect that remains limited in current public datasets. In contrast, the present study focuses exclusively on the DRIVE dataset, which provides high‐quality, manually curated vessel annotations essential for accurate A/V labelling and AVR computation. Despite the restricted dataset size, the proposed Res‐UNet + GCN fusion framework attains a competitive accuracy of 96.3%, demonstrating strong segmentation integrity and stable A/V discrimination. The incorporation of graph‐based topological features allows the model to preserve vessel connectivity and correctly resolve ambiguous branching patterns, contributing to its robust performance even without multi‐dataset training.

Overall, while numerical accuracy values may differ across studies, such comparisons must be interpreted in the context of dataset diversity, annotation availability and experimental design. Under these considerations, the performance of the proposed method remains highly competitive and practically meaningful for hypertensive retinopathy analysis.

#### 4.3.1 Dataset Considerations and Generalisation

Although the proposed method demonstrates competitive performance compared to existing approaches, it is essential to discuss the limitations of the dataset and the model's generalisation capacity. Despite the effectiveness of fundus imaging as a non‐invasive diagnostic tool, publicly available datasets that include reliable artery/vein annotations remain extremely scarce. Most large‐scale retinal datasets provide vessel maps or pathology labels, but very few offer consistent A/V differentiation, which requires expert manual tracing for each major vessel branch. For this reason, the DRIVE dataset was selected for the present study, as it contains high‐quality, manually curated vessel annotations that are essential for meaningful A/V classification and AVR computation. Although the number of samples in DRIVE is limited, several components of the proposed framework mitigate the challenges typically associated with small datasets. First, the preprocessing pipeline standardises illumination conditions and enhances vessel contrast, reducing variability across images. Second, the extensive augmentation strategy incorporating controlled rotations, flips and intensity perturbations exposes the model to a wide range of plausible anatomical and imaging variations, thereby improving generalisation. Third, the graph‐based reasoning employed in the GCN module leverages structural vessel connectivity rather than relying solely on pixel intensities, enabling the model to retain robustness even when trained on a compact dataset. Taken together, these design choices allow the proposed system to generalise beyond the limited number of training samples and maintain consistent performance under realistic clinical imaging conditions. Nonetheless, the scarcity of publicly available A/V datasets highlights an ongoing need for broader annotated resources to support future advancements in this domain.

## 5 Conclusion and Future Work

**FIGURE 18:**
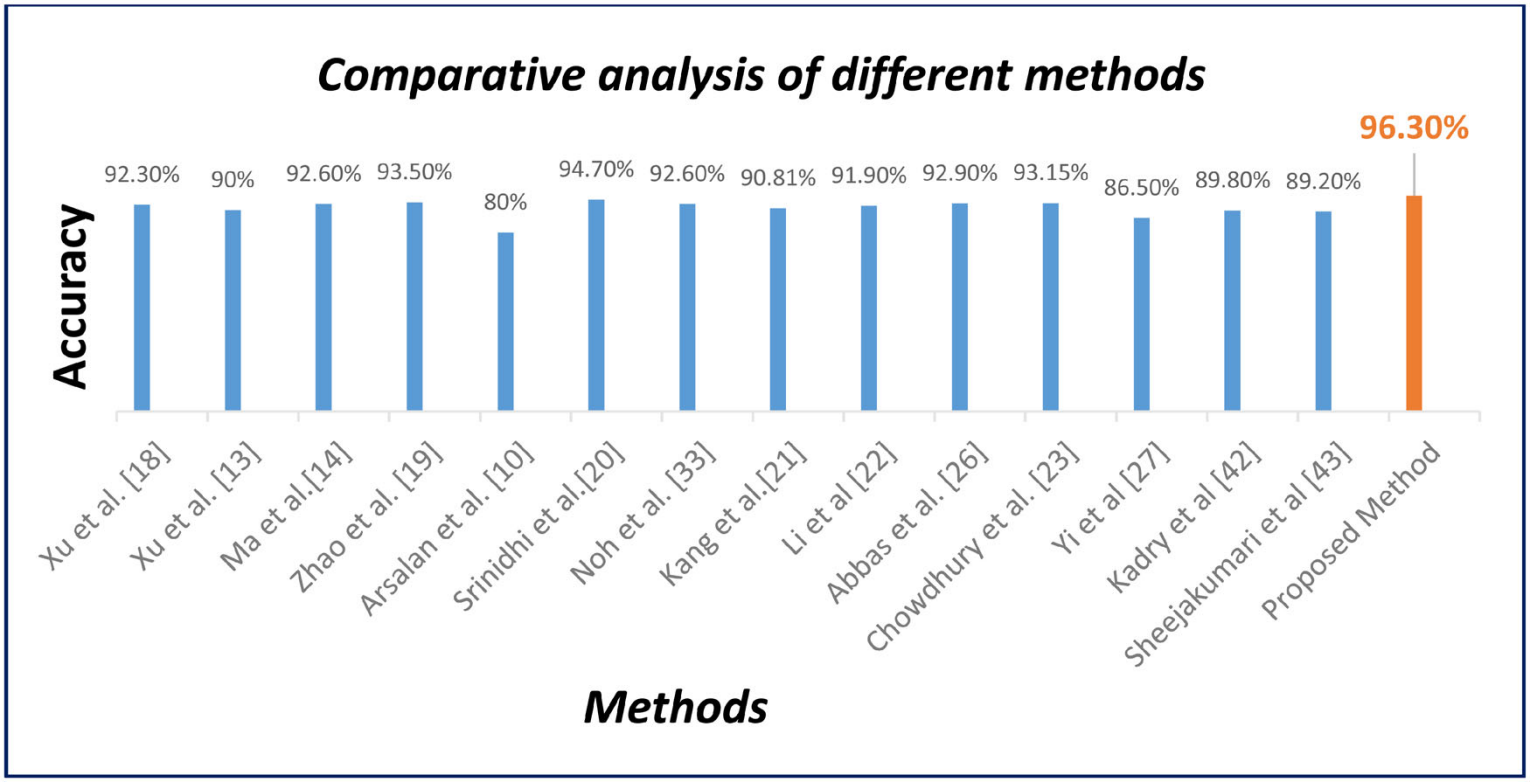
Comparative analysis of different methods.

We have created a deep learning system in this work to identify hypertensive Retinopathy diseases with automatic diagnosis. It has exceptionally high accuracy for identifying HR, as seen in Figure 18. The accuracy of this investigation will be its standard and it will have a major influence on the domains of medical research and ophthalmology, blindness and vision impairment. In the last five years, there has been an increase in research interest in employing CNN to classify AV and HR. However, these algorithms sometimes struggle to accurately segment the AV and classify the HR. Despite their potential, CNN‐based methods have shown limitations in achieving high segmentation accuracy, prompting researchers to seek alternative approaches to address this challenge. This study aims to enhance the accuracy of A/V classification through a novel approach that combines Res‐UNet and GCN models. This method involves extracting vessel features from the Res‐UNet in the image domain and transforming them into a graph representation. The GCN is then applied to learn both Res‐UNet features and vessel architecture features, resulting in improved A/V classification accuracy. This approach is designed to leverage the benefits of both Res‐UNet and GCN, which are powerful tools in image analysis and network modelling to enhance the accuracy of HR classification. For the GCN and Res‐UNet models, we have obtained accuracy values of 0.967 and 0.963, respectively. The suggested approach has been compared with the benchmark approaches. The model may be applied in automated systems in the future that are intended to evaluate vascular morphological and functional changes in retinal and optic disc images. The technique may be evaluated in clinical settings for automated indirect monitoring of intracranial pressure as well as for the early detection and development of disorders which pose a danger to vision, such as vascular occlusions, glaucoma and diabetic retinopathy.

## Author Contributions

Esraa Al Sariera was solely responsible for the conception, methodology design, implementation, experimental evaluation, result analysis and writing of the manuscript.

## Funding

The author has nothing to report.

## Code Availability Statement

Due to institutional restrictions and ongoing related research, the source code associated with this study cannot be made publicly available at this time. Nevertheless, all methodological steps, hyperparameters and configuration settings have been fully documented in the manuscript to support reproducibility. The code will be made available upon reasonable request following the completion of the accompanying research projects.

## Conflicts of Interest

The author declares no conflict of interest.

## Data Availability

The data utilised to support these research findings is accessible online at https://www.kaggle.com/datasets/mehwishmehmood555/drive‐av‐augmentation‐1000img
